# 

**Published:** 1905-01-28

**Authors:** 


### NOTICE TO CORRESPONDENTS.

All MSS., Letters, Books for Review, and other matters intended for the Editor should be addressed The Editor, "The Hospital." The Hospital Buildings, 28 & 29 Southampton Street, Strand, W.C.

The Editor cannot undertake to return rejected MS., even when accompanied by stamped directed envelope.

**Notice to Advertisers and Subscribers.**

All Advertisements, Orders for Copies of The Hospital, and Business Communications should be addressed to **THE MANAGER (Not to the Editor),** The Hospital. 28 & 29 Southampton Street, Strand, London, W.C.

The Cost of Subscription, post free, is as follows:—Per week, 3½d.; per quarter, 3s. 3d.; per half-year, 6s. 6d.; per annum, 13s. Foreign and Colonial:—Per annum, 19s. 6d., payable, in all cases, in advance.

**NOTICE.—Vol. XXXVI. of "The Hospital" (April to September 1904), in handsome cloth binding, is now on sale at the office of this Journal, price 10s. 6d. Cases for binding the Numbers contained in this Volume 2s. Index to Vol. XXXVI., 3½d., post free.**

**The Monthly part for January is now ready. Price 1s., by post, 1s. 3d.**

### Medical Appointments.

DEVONSHIRE HOSPITAL, BUXTON, DERBYSHIRE

ASSISTANT HOUSE SURGEON.

The Committee of Management invite applications for this post. Candidates must be fully qualified and registered.

Salary £70 per annum, with Furnished Apartments, Board (excepting Stimulants), and Laundry.

The appointment will be made for twelve months.

Applications, with recent testimonials, endorsed "Assistant House Surgeon," to be sent to the Secretary at once. Printed regulations will be supplied.

By order,

January 24th, 1905. WILLIAM STEVENSON, Secretary. (1073)

The

### Nurses' Book Page.

**A HANDBOOK FOR NURSES.**
By J. K. WATSON, M.D., M.B., C.M.
New and Revised Edition. Profusely illustrated. 5/- net, 5/4 post free.

**NURSING: Its Theory and Practice.**
Being a complete Text-book of Medical, Surgical, and Monthly Nursing.
By PERCY G. LEWIS, M.D., M.R.C.S., L.S.A.
New Edition. Profusely illustrated.
3/6 post free.

**SURGICAL WARD-WORK AND NURSING.**
By ALEXANDER MILES, M.D.Edin., C.M., F.R.C.S.E.
Second Edition. With particulars and Illustrations of the instruments used in various operations.
3/6 net, 3/10 post free.

**OPHTHALMIC NURSING.**
By SYDNEY STEPHENSON, M.B., F.R.C.S.E.
New and Revised Edition.
3/6 net, 3/10 post free.

**ELEMENTARY ANATOMY AND SURGERY FOR NURSES.**
By WILLIAM McADAM ECCLES, M.S.Lond., M.B., F.R.C.S., L.R.C.P.
2/6 post free.

**ELEMENTARY PHYSIOLOGY FOR NURSES.**
By C. F. MARSHALL, M.D., B.Sc., F.R.C.S.
A valuable help to Physiology for Probationers. Crown 8vo. illustrated, cloth.
2/- post free.

**NURSING IN DISEASES OF THE THROAT, NOSE, AND EAR.**
By P. MacLEOD YEARSLEY, F.R.C.S.Eng., M.R.C.S., L.R.C.P.Lond.
2/6 post free.

**HOSPITAL SISTERS AND THEIR DUTIES.**
By EVA C. E. LÜCKES, Matron, London Hospital. Third Edition.
2/6 post free.

**A COMPLETE HANDBOOK OF MIDWIFERY.**
The Latest and Best Text-Book for the L.O.S.
By J. K. WATSON, M.D.Edin.
143 Illustrations.
6/- net, 6/4 post free.

**A PRACTICAL HANDBOOK OF MIDWIFERY.**

By FRANCIS W. HAULTAIN, M.D.

Illustrated.

6/- net, 6/3 post free.

**PRACTICAL GUIDE TO SURGICAL BANDAGING AND DRESSINGS.**
By WM. JOHNSON SMITH, F.R.C.S.
A handy and useful reference book.
Suitable size for apron pocket.
2/- post free.

**THE EXAMINATION OF THE URINE.**
Compiled for the Use of Nurses.
By J. K. WATSON, M.D., M.B., C.M.
1/- net, 1/1 post free.

**THE NURSES' PRONOUNCING DICTIONARY OF MEDICAL TERMS AND NURSING TREATMENT.**
By HONNOR MORTEN.
New Edition, with phonetic pronunciations.
Edited by MARY I. BURDETT.
2/- post free.

**THE HUMAN BODY.**
Its Personal Hygiene and Practical Physiology.
By B. P. COLTON. Profusely Illustrated.
5/- post free.

**MASSAGE AND MEDICAL GYMNASTICS, including the Swedish and Schott (Nauheim) Movements.**
By AXEL V. GRAFSTROM, B.Sc., M.D.
2/6 post free.

**ART OF MASSAGE.**
By Mrs. CREIGHTON HALE. Second Edition.
6/- post free.

*Publishers of Nursing Text-Books, Charts, Case-Books, &c., of all Descriptions.*

SEND FOR NEW CATALOGUE OF NURSING MANUALS.

**THE SCIENTIFIC PRESS, Ltd.,**

28 and 29 Southampton Street, Strand, London, W.C.

